# Perioperative Oxygen Supply-Demand Balance in Patients Undergoing Hemodialysis With Arteriovenous Fistulas: A Retrospective Observational Study

**DOI:** 10.7759/cureus.81554

**Published:** 2025-04-01

**Authors:** Asuka Kitajima, Yusuke Iizuka, Yuji Hirasaki, Koichi Yoshinaga, Ikumi Sawada, Yuji Otsuka, Masamitsu Sanui

**Affiliations:** 1 Department of Anesthesiology and Critical Care Medicine, Jichi Medical University Saitama Medical Center, Saitama, JPN; 2 Department of Anesthesiology and Critical care Medicine, Jichi Medical University Saitama Medical Center, Saitama, JPN; 3 Department of Anesthesiology, Saiseikai Utsunomiya Hospital, Utsunomiya, JPN; 4 Department of Anesthesiology and Critical Care Medicine, Jichi Medical University, Shimotsuke, JPN

**Keywords:** cardiac surgery, end-stage renal disease, hemodialysis, mixed venous oxygen saturation, oxygen supply-demand balance

## Abstract

Background: Patients with end-stage renal disease on hemodialysis (HD) undergoing cardiac surgery face increased risks. Mixed venous saturation (SvO_2_) is an important parameter representing the systemic oxygen supply-demand balance. However, interpreting SvO_2_ in HD patients may be challenging due to arteriovenous fistulas. The literature on these issues is lacking. This study aimed to investigate the change in SvO_2_ in HD patients by comparing those in non-HD patients perioperatively.

Methodology: From April 1, 2019, to March 31, 2020, 39 patients undergoing cardiac surgery with pulmonary artery catheters, 18 with and 21 without HD, were identified. The cardiac index (CI) and SvO_2_ were extracted from patient records, and the oxygen delivery index (DO_2_I) was calculated before surgery (T0), on intensive care unit (ICU) admission (T1), 24 hours (T2), and 48 hours (T3) after ICU admission. A linear mixed effects model was applied for repeated measures analyses.

Results: T0 CI was significantly higher in the HD group (2.5 ± 0.5 vs. 2.0 ± 0.5 L/minute/m^2^, mean ± SD, *P* = 0.003) and increased significantly over time in both groups, without an interaction effect (*P* for interaction = 0.12). T0 SvO_2_ did not differ between groups (72 ± 10% vs. 72 ± 5%, *P* = 0.97) and decreased over time, more evidently in the non-HD group (*P* for interaction = 0.016). DO_2_I was similar in both groups perioperatively.

Conclusions: SvO_2_ tended to be higher in the HD group perioperatively. If SvO_2_ in HD patients is similar to that in non-HD patients, this may mean that the oxygen supply-demand balance is disturbed.

## Introduction

The kidneys play essential roles in homeostasis, specifically in removing water-soluble waste, regulating blood pressure, erythropoiesis, and ion and bone matrix metabolism [1]. End-stage renal disease (ESRD), in which these kidney functions are lost, poses a tremendous threat to life, and patients with ESRD, therefore, require either renal replacement therapy or a kidney transplant to survive. 

Hemodialysis (HD) is a renal replacement therapy in which blood is drawn, dialyzed, and pumped back into the patient's bloodstream. An arteriovenous fistula is created to facilitate regular HD. While HD extends the life expectancy of patients with ESRD, these patients have a high risk of developing cardiovascular diseases such as hypertension, congestive heart failure, and vascular and valvular calcification [2,3]. The literature shows that patients with ESRD often develop coronary artery stenosis, aortic valve stenosis (AS) [3,4], or both, which require surgical intervention. Due to their high risk of morbidity and mortality [5,6], patients with ESRD undergoing cardiac surgery need close perioperative oxygen supply-demand balance monitoring to ensure successful outcomes [7].

Pulmonary artery catheters (PACs) can be used for close perioperative oxygen supply-demand balance monitoring. PACs allow for cardiac output (CO) measurements and mixed venous saturation (SvO_2_), representing the systemic oxygen supply-demand balance. However, caution is required when interpreting these parameters. Earlier research has shown that CO gradually increases in patients with ESRD after creating an arteriovenous fistula [8-10] and that such patients exhibit a higher blood oxygen saturation in the superior vena cava (ScvO_2_) than in healthy populations [11]. Moreover, a low ScvO_2_ during dialysis is associated with increased complications and mortality [12].

Despite the altered hemodynamic parameters in dialysis patients compared to non-dialysis patients, there is currently no research investigating the behavior of these parameters in dialysis patients during the perioperative period, a time when hemodynamics is likely to change. Consequently, the appropriate target values for perioperative management of these parameters in HD patients remain unclear, even though they represent a high-risk patient group.

 This study aims to evaluate perioperative hemodynamic changes in HD versus non-HD patients undergoing cardiac surgery, focusing on SvO₂ as a marker of oxygen supply-demand balance. Moreover, we investigated how oxygen supply-demand balance parameters change during the perioperative period.

## Materials and methods

The study protocol was approved by the institutional review board of our hospital (Jichi Medical University Bioethics Committee for Clinical Research, Saitama Medical Center, S22-123, February 16, 2023). The research was conducted in accordance with the Declaration of Helsinki. Informed consent was obtained from the patients by an opt-out method. The requirement for consent to publish these findings was waived due to the retrospective nature of our analyses and the use of anonymized data.

We retrospectively reviewed our hospital's medical records and extracted the data of adult patients who underwent cardiovascular surgery with PAC placement between April 1, 2019, and March 31, 2020. During this period, the PAC placement in cardiovascular surgery was indicated in the following patients according to our institutional criteria: (1) those on HD undergoing cardiac surgery under cardiopulmonary bypass or off-pump coronary artery bypass grafting (OPCABG), and (2) those over 70 years of age with chronic kidney disease (estimated glomerular filtration rate <60 mL/minute) undergoing aortic valve replacement for AS.

All patients received general anesthesia and tracheal intubation. Standard monitoring, including an arterial line, transesophageal echocardiography, and a PAC, was performed. The general intraoperative target values for cardiovascular surgery were cardiac index (CI) > 2.5 L/minute/m^2^, mean arterial pressure > 65 mmHg, hemoglobin (Hb) > 8.0 g/dL, SvO_2_ > 65%, and plasma lactate level < 4 mmol/L. Intraoperatively, fluid and blood transfusions or catecholamines were administered at the discretion of the attending anesthetist. During cardiopulmonary bypass, blood concentration was not usually performed at our facility. All patients were admitted to the intensive care unit (ICU) after surgery and extubated when their hemodynamics were stable.

A PAC with thermal filament (Swan-Ganz thermodilution catheter CCO/CEDV, 774F75, Edwards Lifesciences, Irvine, CA) was placed after general anesthesia was induced, and CO and SvO_2_ levels were continuously monitored using a Vigilance II hemodynamic monitor (Edwards Lifesciences). SvO₂ calibration was performed using samples taken from the pulmonary artery, both early after insertion of the PAC and when the patient entered the ICU. Subsequent calibration was dependent on the ICU doctor.

The oxygen supply-demand balance parameters were collected from each patient’s records at four different time points. The parameters included the SvO_2_, CI, Hb levels, PaO_2_/FiO_2_ ratio, base excess (BE), and lactate levels. As SvO_2_ is affected by Hb and arterial oxygen saturation (SaO_2_), perioperative changes in Hb and SaO_2_ were also collected. Using the obtained parameters, the oxygen delivery index (DO_2_I) was calculated according to the following equation: DO_2_I = (Hb * 1.34 * SaO_2_ * CO + 0.003 * PaO_2_) / BSA, where SaO_2_, PaO_2_, and BSA stand for arterial oxygen saturation, partial pressure of arterial oxygen and body surface area, respectively. We also collected data on the perioperative doses of catecholamines (noradrenaline, dobutamine, milrinone) administered and fluid balance after surgery. Data were collected before surgery (T0), on admission to the ICU (T1), 24 hours (T2), and 48 hours (T3) after admission. In addition, to determine whether the changes in CO observed in HD patients were due to increases in left ventricular volume, we also extracted the following parameters from the preoperative echocardiography: left ventricular end-diastolic diameter, left ventricular end-systolic diameter, left ventricular end-diastolic volume, left ventricular end-systolic volume, and ejection fraction. The ejection fraction was estimated by using the modified Simpson’s method. Furthermore, to investigate the effect of tricuspid regurgitation on the measured values, we also collected preoperative tricuspid regurgitation peak gradient values from the preoperative echocardiography.

Statistical analysis

Normally distributed variables were presented as the means ± standard deviations, and variables with a skewed distribution as the medians and interquartile ranges. Missing data were not substituted. Categorical variables were presented as numbers and percentages of patients. Student t-tests, the Mann-Whitney U test, and Chi-square tests were used to compare groups as applicable. The null hypothesis tested was that there would be no difference in CI, SvO_2_, and other changes between the HD and non-HD groups in the perioperative period. A linear mixed-effects model was applied for repeated measures analyses with groups (HD or non-HD) as the independent variable, each measured parameter (CI, SvO_2_, Hb, PaO_2_/FiO_2_ ratio, BE, lactate levels, and DO_2_I) as the dependent variable, and the individual as the random effect. A type III analysis of variance with Satterthwaite’s method was performed to calculate P for interaction to examine whether there was an interaction effect between the groups and time course. Based on a mean difference of 5% for SvO_2_, a standard deviation of 5%, a significance level of 0.05, and a power of 0.8 for SvO_2_ at T0, the required sample size was estimated at 15 patients per group. In the absence of previous studies, we set a clinically significant difference of 5%. A Bonferroni adjustment was applied to repeated measured parameters. All statistical analyses were performed using R version 4.1.2 and EZR software [13]. Linear mixed-effects models were estimated using the “lme4” package in R software. A sensitivity analysis was not performed.

In preparing this manuscript, we followed the Strengthening the Reporting of Observational Studies in Epidemiology (STROBE) checklist.

## Results

We identified 39 patients who underwent cardiovascular surgery with PAC placement during the study period. Of these, 18 patients receiving HD were assigned to the HD group. The other 21 patients did not receive HD and were assigned to the non-HD group. All patients in the HD group had an arteriovenous fistula in the upper extremity to maintain HD. Patient characteristics are summarized in Table 1. All patients in the non-HD group had AS. For the non-HD patients, only AS patients had routine CO measured by PAC perioperatively, as described in the Methods section. Therefore, five patients in the HD group underwent OPCABG, while all patients in the non-HD group underwent on-pump procedures. The two groups had no differences in left ventricular volume, ejection fraction, tricuspid regurgitation peak gradient, catecholamine doses, or fluid balance. Intraoperative red cell transfusion was more frequent in the HD group.

**Table 1:** Patient characteristics. Data are presented as the means ± SDs or medians (interquartile ranges). Complex procedures include two or more procedures on valves and coronary arteries. HD, hemodialysis; BSA, body surface area; LVDd, left ventricular end-diastolic diameter; LVDs, left ventricular end-systolic diameter; LVEDV, left ventricular end-diastolic volume; LVESV, left ventricular end-systolic volume; SD, standard deviation; TRPG, tricuspid regurgitation peak gradient; n.a., not available; CPB, cardiopulmonary bypass; AVR, aortic valve replacement; OPCABG, off-pump coronary artery bypass graft; T0, T1, T2, and T3 represent before surgery, on admission to the intensive care unit, and at 24 and 48 hours after admission, respectively.

		HD (*n *= 18)	Non-HD (*n *= 21)	*P*-value
Preoperative states				
Age (years)		74 (64-77)	77 (72-78)	0.05
Male, *n* (%)		9 (50)	11 (52)	1
BSA (m^2^)		1.6 ± 0.2	1.6 ± 0.2	0.55
Dialysis history (years)		6 (3.3-16.3)	n.a.	n.a.
LVDd (mm)		50 (47-55)	45 (42-49)	0.19
LVDs (mm)		30 (27-40)	28 (25-31)	0.21
LVEDV (mL)		89.7 (78.8-297.9)	78.2 (67.8-126.6)	0.68
LVESV (mL)		29.8 (27.4-32.6)	24.2 (20.6-52)	0.75
Ejection fraction (%)		68 (49.5-71)	69 (65-71)	0.53
TRPG (mmHg)		24.4 ± 10.9	25.0 ± 9.7	0.87
EuroSCORE II (%)		2.6 (2.0-3.5)	4.0 (1.4-4.9)	0.61
Procedure, *n* (%)				
AVR		9 (50)	10 (48)	-
OPCABG		5 (28)	0	-
Complex		4 (22)	11 (52)	-
Length of surgery (minutes)		262 (231-329)	255 (204-300)	0.61
Length of CPB (minutes)		125 (86-147) (*n *= 13)	130 (95-167)	0.86
Red cell transfusion (unit)				
Intraoperative		5 (4-6)	0 (0-4)	0.001
Postoperative		0 (0-0)	0 (0-0)	0.51
Doses of catecholamines (microgram/kg/minute)				
Noradrenaline	T0	0 (0-0)	0 (0-0)	0.38
	T1	0 (0-0)	0 (0-0)	n.a.
	T2	0 (0-0)	0 (0-0)	0.30
	T3	0 (0-0)	0 (0-0)	0.30
Dobutamine	T0	0 (0-0)	0 (0-0)	0.30
	T1	0.6 (0-2.7)	0 (0-1.5)	0.34
	T2	0 (0-3.5)	0 (0-1.6)	0.31
	T3	0 (0-2.2)	0 (0-1.1)	0.52
Milrinone	T0	0 (0-0)	0 (0-0)	n.a.
	T1	0 (0-0)	0 (0-0)	0.63
	T2	0 (0-0)	0 (0-0)	0.67
	T3	0 (0-0)	0 (0-0)	0.20
Fluid balance after surgery (mL)		4047 ± 1644	4229 ± 970	0.67

**Figure 1:**
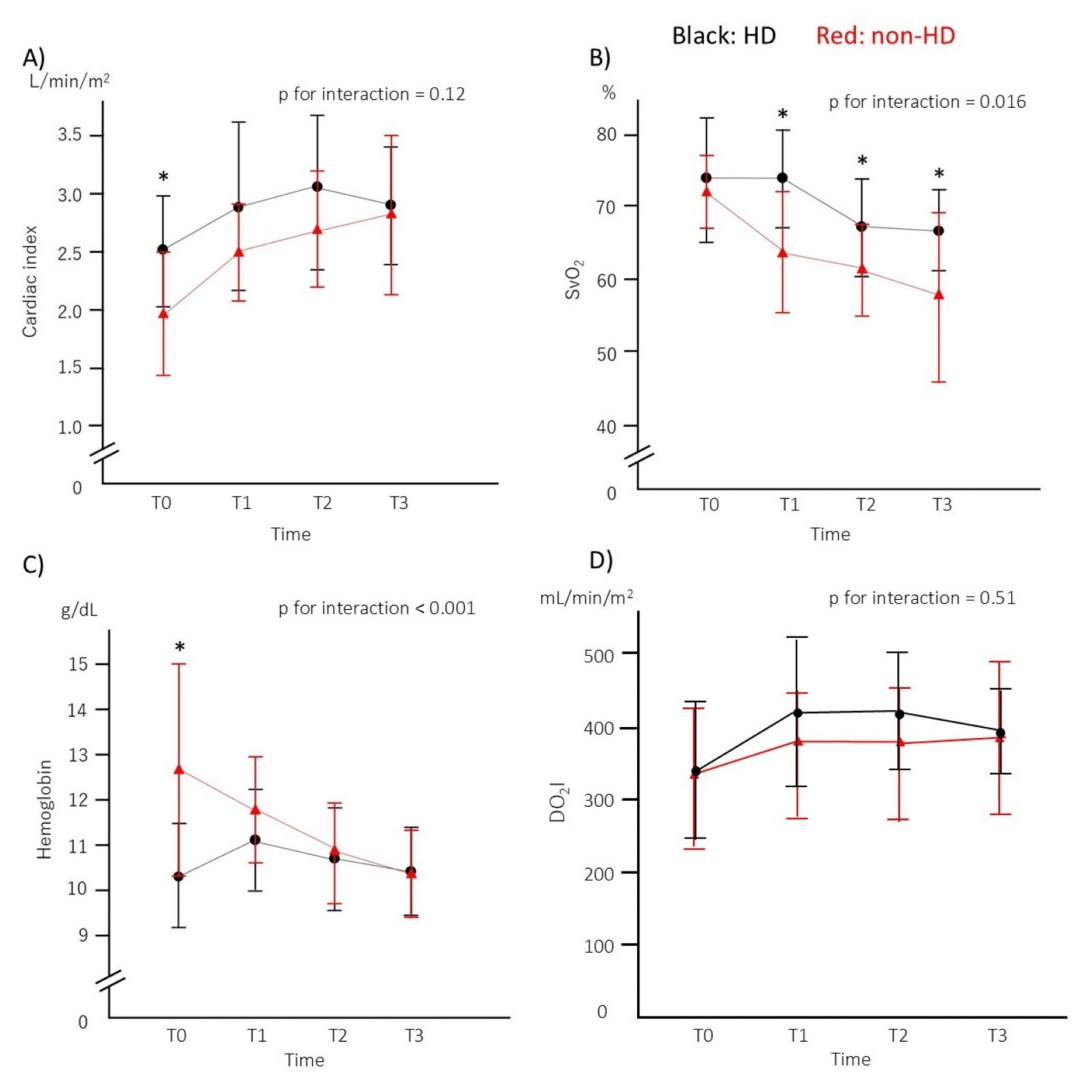
Time course of parameters. Time course of the cardiac index, mixed venous oxygen saturation (SvO_2_), hemoglobin levels, and oxygen delivery index (DO_2_I) in the hemodialysis group (HD, black) and the non-hemodialysis group (non-HD, red). T0, T1, T2, and T3 represent mean values before surgery, on admission to the intensive care unit, and at 24 and 48 hours after admission, respectively. Error bars indicate standard deviations. Asterisks (*) show a significant difference between groups (*P* < 0.0125).

At T0, the mean CI was significantly greater in the HD group than in the non-HD group (2.5 ± 0.5 vs. 2.0 ± 0.5 L/minute/m^2^, *P* = 0.003). Although the CI significantly increased over time in both groups (*P* < 0.001), there was no interaction effect between groups and time (*P* for interaction = 0.12; Figure 1, top left). SvO_2_ levels did not differ between the groups at T0 (72 ± 10 vs. 72 ± 5 %, *P* = 0.97). Subsequently, both groups showed a significant decrease in SvO_2_ over time (*P* < 0.001). In particular, the magnitude of the decrease was significantly greater in the non-HD group than in the HD group (*P* for interaction = 0.016; Figure 1, top right). Hb levels were significantly lower in the HD group at T0 (10.3 ± 1.1 vs. 12.7 ± 2.3 g/dL, *P* < 0.001). Both groups showed a significant decrease in Hb over time (*P* < 0.001). Although there appears to be an interaction effect between the groups and time course (*P* for interaction < 0.001; Figure 1, bottom left), the impact of red cell transfusion is likely to be significant, as the HD group received significantly more transfusions from T0 to T1 (Table 1). In DO_2_I, there was no difference between the two groups at T0 (340 ± 94.0 vs. 334 ± 89.0 mL/minute/m^2^, *P* = 0.85), and no interaction was observed (*P* for interaction = 0.51, bottom right). The time course of the PaO2/FiO2 ratio was similar between the groups. Lactate and BE levels peaked at T1 and then returned toward baseline values.

## Discussion

We conducted this study to investigate if there are any differences in parameters related to the oxygen supply-demand balance between patients receiving and not receiving HD undergoing cardiac surgery. In the present study, mean preoperative CI was significantly greater in the HD group and increased significantly over time in both groups, but there was no interaction effect between groups and time course. For SvO_2_, the groups did not differ preoperatively. Thereafter, SvO_2_ decreased significantly over time in both groups, with the magnitude of the decrease being significantly greater in the non-HD group than in the HD group. For Hb levels, the effect of blood transfusion was greater in the HD group, and DO_2_I was similar in both groups perioperatively.

As mentioned earlier, high CO and ScvO_2_ in hemodialysis patients have been reported, which may be due to arteriovenous fistula [7-10]. This may be because arterial blood with high oxygen saturation enters the venous side through the arteriovenous fistula. Similarly, HD patients may have higher SvO_2_ than healthy individuals, but no reports have specifically examined SvO_2_. Therefore, the value of SvO_2_ that should be used as an indicator in HD patients is unknown. In addition to the underlying arteriovenous shunt effect, anemia may contribute to increased CI in hemodialysis patients [14]. These changes may further complicate the assessment of SvO2 in HD patients. Furthermore, although there is no SvO_2_ target value specific to cardiac surgery, SvO2 >65% is generally considered acceptable at our facility. However, it is currently unclear whether a similar value should be used in HD patients.

From this study, we obtained two major findings. First, HD patients tended to have a higher CI from T0 to T2 than the non-HD group. The difference in mean CI was approximately 500 mL/minute/m^2^. We assume this was more likely due to arteriovenous fistulae since it is a common shunt flow rate in patients on chronic intermittent dialysis [15]. However, at T3, CI and Hb levels were similar in both groups. Theoretically, the CI should also be greater in the HD group at T3 because of the arteriovenous fistulae. This finding could be due to the small sample size. Although the difference in the amount of water removed may have been involved, we could not determine the exact reason for this finding because this was not investigated in this study.

Second, SvO_2_ was consistently higher in the HD group postoperatively, although the DO_2_I calculated from the CO measured by PAC was almost the same in both groups postoperatively. This suggests that oxygen consumption (VO_2_) was lower in the HD group than in the non-HD group after surgery. VO_2_ in HD patients was reported to be lower than in healthy volunteers. Low VO_2_ in HD patients may be explained by altered microvascular function or mitochondrial insufficiency in HD patients, which causes impaired oxygen extraction or oxygen consumption rather than oxygen delivery [16,17]. According to Stray-Gundersen et al., electron microscope images of the peripheral blood vessels of dialysis patients show thickened capillary endothelium and electron-dense deposits in the interstitial space, suggesting that oxygen diffusion and waste removal may be impaired [16]. Impaired oxygen utilization may also disrupt the oxygen supply-demand balance. Further studies measuring direct VO₂ or tissue oxygenation indices (e.g., near-infrared spectroscopy) may help to consider these mechanisms. In contrast, SvO_2_ did not differ between the two groups preoperatively. There was also no difference in DO_2_I, although DO_2_I at T0 was lower in both groups compared with healthy individuals [18]. This may be due to the induction of general anesthesia in addition to patients' cardiac function. The fact that SVO_2_ and DO_2_I showed similar values means that VO_2_ was also equivalent in both groups at that time point. Since both groups were under general anesthesia, the difference in VO_2_ might be minimized [19].

Due to the low VO_2_ in the HD group, the SVO_2_ may be higher in the HD group in the resting and awake states. After the induction of general anesthesia, the difference in VO_2_ was minimized, and if DO_2_ was the same, SVO_2_ would be the same in both groups. After surgery, SvO_2_ was higher in the HD group. This course is important and can be explained by the difference in VO_2_, but attention should be paid when assessing DO_2_ in HD patients. Since the shunt blood flow does not contribute to systemic oxygen transport, the *effective* DO_2_ that contributes to systemic circulation could be lower than the apparent DO_2_ in HD patients. If there is a decline in cardiac function due to long-term dialysis [20], it will lead to a further decline in effective DO_2_, worsening the oxygen supply-demand balance.

It is essential to maintain effective DO_2_. Since the HD group was thought to usually have a higher SVO_2_ than non-HD patients, if SVO_2_ was the same as in the non-HD group, there was a possibility that effective DO_2_ was decreasing, and the oxygen supply-demand balance was likely to be disrupted. If there is a situation that increases VO_2_ like shivering or agitation, it should be corrected immediately, and at the same time, intervention to increase DO_2_ should be started as soon as possible. Treating SVO_2_ in HD patients in the same way as non-HD patients is considered dangerous, and it is probably better to set the target value higher than usual.

The strength of this study is that no other study has compared the trends over time of oxygen supply-demand balance parameters, including SvO_2_, in HD and non-HD patients, but it also has the following limitations. First, this study was retrospective and had a small sample size. In this study, we analyzed patients who had undergone cardiovascular surgery with PAC, dividing them into two groups: those who received HD and those who did not. However, the control group was heterogeneous. This large heterogeneity might affect the results as we did not match the surgical procedure due to the small sample size. Based on the results of this study, it will be necessary to consider increasing the homogeneity of the patient background or increasing the number of patients through multicenter studies in the future. Altogether, the sample size was small, and robust conclusions could not be drawn.

Second, catecholamine doses and fluid balance, which affect DO_2,_ were not adjusted due to insufficient sample size. However, there were no significant differences between the two groups. The influence of the difference of those factors might be minimal.

Third, we did not measure VO_2_ in this study. It is unclear how VO_2_ affected each value. Furthermore, whether SvO_2_-guided management impacts postoperative outcomes for HD patients undergoing cardiac surgery remains unknown; this question warrants further study.

Fourth, although SvO_2_ data was collected using the Vigilance II hemodynamic monitor, there was no clear protocol for the timing of calibrations after admission to the ICU. As a result, the accuracy of the obtained values might not be enough. In addition, it may have been possible to obtain more reliable values using velocity time integral or stroke volume from echocardiography rather than continuous CO measurement. However, continuous CO measurement has been reported to be as accurate as other methods with echocardiography [21], so we believe that measurement with continuous CO is reasonable.

## Conclusions

HD patients tended to have a higher CI than the non-HD group, but the DO_2_I was almost the same in both groups perioperatively. SvO_2_ in HD patients undergoing cardiac surgery may be higher than in non-HD patients due to suppressed VO_2_. If SvO_2_ in HD patients is similar to that in non-HD patients, this suggests that the oxygen supply-demand balance was already disturbed. The interpretation of SvO_2_ values requires caution in patients receiving HD. Further investigation with a larger sample size is required to find the appropriate target values for perioperative management of these parameters in patients receiving HD.
